# Some of the Nerve Remedies

**Published:** 1900-10

**Authors:** Edward C. Briggs

**Affiliations:** Boston, Mass.


### SOME OF THE NERVE REMEDIES.[1]

BY DR. EDWARD C. BRIGGS, BOSTON, MASS.

This is the subject as put down by the master of ceremonies, Dr. Cook. It is a very large one, and I do not wish to take your time by attempting to cover it. I will simply confine myself to the use, not of the remedies with which you are all familiar, but to the coal-tar products in their application as remedies to help in relieving pain where surgery is delayed or impossible at the time. It is sometimes necessary that the dentist should give his patients medicine to help them while making a diagnosis of the case, and if you will familiarize yourselves with a few drugs, you will find that

[1] Read before the Alumni of the Harvard Dental School on Alumni Day, June 25, 1900.

it is of great benefit to use them. Morphine and the other alkaloids of opium have always been shunned by dentists. Our experience has been that the patient after taking these drugs was rarely helped and almost always put into a condition of general malaise, which made other treatment impossible or, at least, difficult.

If a patient is threatened with alveolar abscess, or in cases where it is very difficult to make a diagnosis for twenty-four or forty-eight hours, it is a great comfort that you can give some drug which is positively safe and will insure a good night's rest. Germany is the great producer of these coal-tar products, both for medicine and dye-stuffs. They are proprietary drugs, and for that reason a great many unconsciously class them with patent medicines. But Germany simply controls their manufacture. It is all right to use these drugs, and they should not be classed with the "quack" medicines. These drugs are classed as antipyretics and analgesics, particularly for the fifth pair of nerves.

Antipyrin was discovered in 1884. The action of this is sure, but it has caused nausea and even collapse; and therefore, because it was used in large doses, it became a bugbear to the dentist. In 1887 we found acetanilide, derived from aniline, to be better and safer, and a combination of it with bicarbonate of soda and caffeine (antikamnia) is very efficacious and apparently safe.

Phenacetin has been found to have, in some hands, the effect of producing cyanosis and the diminishing of the urine. It has had great use among the laity. It produces severe prostration in some cases, and depression follows its use and does not always relieve pain. From it the relief is not so decided as some of the others. Lactophenin was discovered in 1893 and used with success.

In 1895 Biechler produced a paraphenitidin derivative which is named kryofine. This combination produces decidedly different effects from phenacetin. Much can be said in favor of this drug. It is a febrifuge and antineuralgic. Ammonal is a product of the amido-benzine series, and is a phenylacetamide, in combination with ammonia to strengthen the heart. These drugs will be found in small doses perfectly safe, and by repeating the dose one can control the kind of pain which dentists are called upon to treat.

I repeat, in many cases where you find you must do something and it is not the time for surgical interference, as the diagnosis is not clear, these drugs given in three-grain doses and repeated every half-hour until three, four, or six doses have been taken, will almost invariably give the required relief. In alveolar abscess and pulpitis this treatment is efficacious.

It might be well to speak of one of the newer local anæsthetics. Orthoform, a methyl ether compound of amido-oxybenzoic acid, can be administered internally, one-half gramme to one gramme several times a day. It is non-toxic, and the anæsthesia is slow and progressive. This makes a very satisfactory preparation to put in in cases of exposed pulp where the pain is severe. It will quiet the pulp, and is also a good thing to put into the socket of a tooth after extraction. Sometimes there will be extreme pain after extraction and a violent neuralgic inflammation set in, particularly in cases of the third molar, lower, and you will find this to be most effectual.

In this connection it is well to remind you also, in the treatment of neuralgia, where you suspect that the tooth or the teeth are not at the bottom of it, although the physician declares it must be so, you should always suggest that he try doses of quinine and see if there is not malaria connected with the matter.

